# Ex Vivo Antioxidant and Cholinesterase Inhibiting Effects of a Novel Galantamine–Curcumin Hybrid on Scopolamine-Induced Neurotoxicity in Mice

**DOI:** 10.3390/ijms232314843

**Published:** 2022-11-27

**Authors:** Rumyana Simeonova, Mariyana Atanasova, Georgi Stavrakov, Irena Philipova, Irini Doytchinova

**Affiliations:** 1 Faculty of Pharmacy, Medical University of Sofia, 1000 Sofia, Bulgaria; 2 Institute of Organic Chemistry with Centre of Phytochemistry, Bulgarian Academy of Sciences, 1113 Sofia, Bulgaria

**Keywords:** Alzheimer’s disease, acetylcholinesterase, butyrylcholinesterase, oxidative stress, curcumin, galantamine, catalase, superoxide dismutase, glutathione peroxidase, malondialdehyde, reduced glutathione

## Abstract

Oxidative stress is an essential factor in the development and progression of Alzheimer’s disease (AD). An excessive amount of reactive oxygen species (ROS) induces the peroxidation of lipid membranes, reduces the activity of antioxidant enzymes and causes neurotoxicity. In this study, we investigated the antioxidant and cholinesterase inhibitory potential of a novel galantamine–curcumin hybrid, named **4b**, administered orally in two doses (2.5 mg/kg and 5 mg/kg) in scopolamine (SC)-induced neurotoxicity in mice. To evaluate the effects of **4b**, we used galantamine (GAL) (3 mg/kg) and curcumin (CCN) (25 mg/kg) as positive controls. Ex vivo experiments on mouse brains showed that the higher dose of **4b** (5 mg/kg) increased reduced glutathione (GSH) levels by 46%, catalase (CAT) and superoxide dismutase (SOD) activity by 57%, and glutathione peroxidase (GPx) activity by 108%, compared with the SC-treated group. At the same time, **4b** (5 mg/kg) significantly reduced the brain malondialdehyde (MDA) level by 31% and acetylcholinesterase (AChE) and butyrylcholinesterase (BChE) activities by 40% and 30%, respectively, relative to the SC-impaired group. The results showed that **4b** acted as an antioxidant agent and brain protector, making it promising for further experimental research in the field of neurodegenerative diseases.

## 1. Introduction

Alzheimer’s disease (AD) is among the most significant diseases of our time, with a tendency to become a pandemic among older adults [1]. AD is characterized by the accumulation of extracellular amyloid plaques and intraneural neurofibrillary tangles, progressive acetylcholine (ACh) deficiency and brain destruction. As a typical neurodegenerative disease, AD causes a gradual loss of memory and cognitive functions, followed by the failure of physical functions in the final stages [2].

Oxidative stress has been found to be an essential factor in the development and progression of AD through the peroxidation of biomolecules, the amyloid-beta (Aβ) redox potential of metal ions and mitochondrial damage. The brain requires increased amounts of oxygen to perform its synaptic functions and is highly sensitive to oxidative stress [3]. Excessive reactive oxygen species (ROS) induce lipid membrane peroxidation and cause neurotoxicity affecting hippocampal plasticity, thus directly contributing to the pathogenesis of AD [4]. Since ROS mediate neurotoxicity in AD, the invention of compounds with dual action, such as antioxidants and as cholinesterase inhibitors for neuroprotection, is a more desirable strategy for clinical use. Furthermore, there is evidence [5] that the dual cholinesterase inhibition of acetylcholinesterase (AChE) and butyrylcholinesterase (BChE) has a better effect on the control of AD.

Scopolamine (SC), a nonselective muscarinic cholinergic antagonist, induces neurotoxicity by reducing the level of the major neurotransmitter ACh and then increasing AChE activity in the brain [6]. It also stimulates the generation of ROS, thereby inducing oxidative stress manifested by increased lipid peroxidation and impairment of antioxidant defense mechanisms. SC-induced experimental models of AD can mimic the oxidative stress damage in human AD development; thus, they have been used as a model in anti-AD drug screening in many studies [7,8,9].

**Figure 1:**
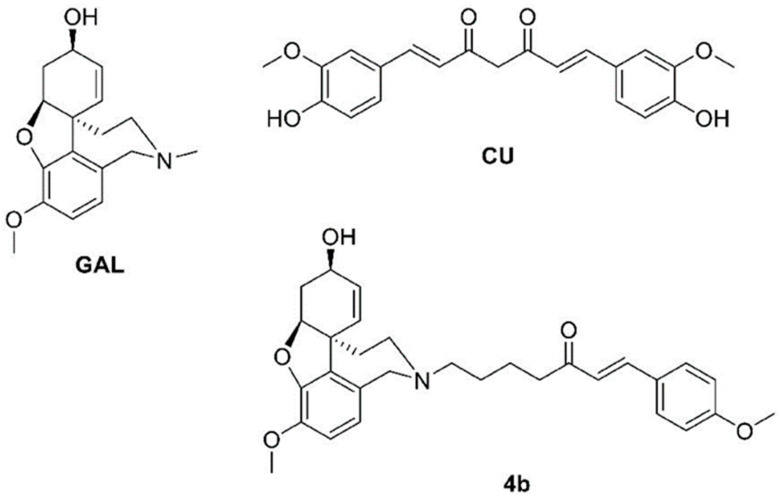
Structures of galantamine (GAL), curcumin (CCN) and their hybrid **4b**.

We recently designed a combinatorial library of hybrid molecules combining fragments from the antioxidant curcumin (CCN) and the AChE inhibitor galantamine (GAL). The hybrids were screened for optimal ADME and blood–brain permeability properties and were docked to AChE [10]. The best performing molecules were synthesized and tested for neurotoxicity and AChE inhibition in vitro. Among them was a molecule named **4b**, which showed anti-AChE activity 186 times higher than GAL (Figure 1). This compound was nontoxic at short-term administrations of 2.5 and 5 mg/kg in mice and demonstrated powerful antioxidant activity that was 10 times more potent than the positive control butylhydroxytoluene (BHT) in an ABTS assay [11]. Here, we describe the ex vivo evaluation of the antioxidant and anticholinesterase effects of the hybrid **4b** on SC-induced neurotoxicity in mice. Since the present study is an extension of our previous study on **4b** [10,11], we prefer to keep the same compound ID. The parent compounds—GAL and CU—were used in this study as reference compounds. The antioxidant activity was measured on the enzymes catalase (CAT), superoxide dismutase (SOD) and glutathione peroxidase (GPx), which are involved in the neutralization of ROS [12,13]. In addition, malondialdehyde (MDA) and reduced glutathione (GSH) levels were measured. MDA is a specific marker for lipid peroxidation [14], while GSH is an important endogenous cellular protector and antioxidant [15]. The anticholinesterase activity was measured on both AChE and BChE.

## 2. Results

Alzheimer’s type dementia was induced by an *ip* injection of SC 3 mg/kg to male mice for a period of 14 days, according to the protocol described in the Materials and Methods section. After the last treatment, brain homogenates were collected and used to assess the anticholinesterase activities of the tested compounds GAL, CCN and **4b** in two doses (low dose (LD) (2.5 mg/kg) and high dose (HD) (5 mg/kg)) on AChE and BChE, and their antioxidant activities on catalase (CAT), superoxide dismutase (SOD), glutathione peroxidase (GPx), malondialdehyde (MDA) and reduced glutathione (GSH).

### 2.1. Antioxidant Activities on Enzymes and Biochemical Markers of Oxidative Stress

The antioxidant activities of GAL, CCN and **4b** are given in Table 1. The antioxidant enzymes CAT, SOD and GPx are involved in the neutralization of ROS in the brain. When administered alone, GAL, CCN and **4b** did not significantly affect the enzyme activities, while SC reduced them by 37%, 39% and 51% for CAT, SOD and GPx, respectively. The effects on GSH were also opposite: while GAL, CCN and both doses of **4b** increased the levels by 9%, 28%, 30% and 40%, respectively, SC decreased them by 31% compared with the control. Regarding MDA levels, GAL, CCN and both doses of **4b** did not significantly change them, while SC increased MDA levels by 85% compared with the control group.

The 14-day coadministration of SC with GAL, CCN and **4b** eliminated the neurotoxic effects of SC. The enzyme activities and the GSH and MDA levels were close to these of the control group, demonstrating a full antioxidant protection by GAL, CCN and **4b**. Compared with the SC group, GAL increased the level of GSH by 52%; the activity of CAT, SOD and GPx by 35%, 44% and 60%, respectively; and decreased the amount of MDA by 25%. CCN significantly increased the activity of the investigated enzymes CAT, SOD and GPx by 60%, 58% and 83%, respectively; the level of GSH by 68%; and decreased the amount of MDA by 31% compared with the SC control group. The same trend was observed for both doses of **4b** in combination with SC. The changes caused by **4b** were as follows: administered at a dose of 2.5 mg/kg, it significantly increased the amount of GSH by 32%; the activities of CAT, SOD and GPx by 52%, 51% and 94%, respectively; and reduced the amount of MDA by 26%. The higher dose of **4b** produced similar changes: GSH level increased by 46%, CAT and SOD activity increased by 57%, GPx increased by 108% and MDA level decreases by 31% compared with the SC group.

### 2.2. Assessment of Cholinesterase Inhibition

**Figure 2:**
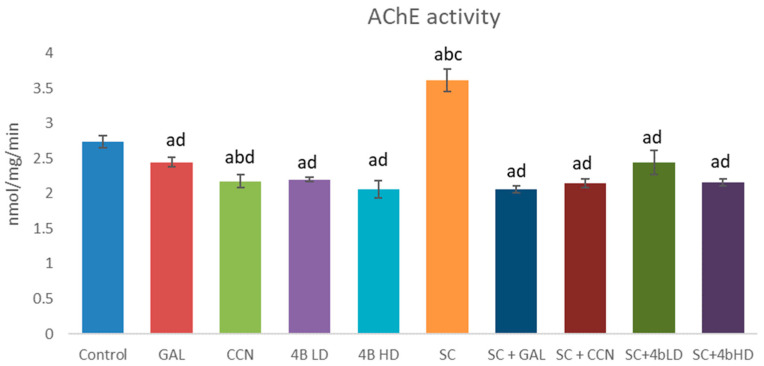
AChE activity measured over 10 min in brain homogenates from mice treated for 14 days with GAL, CCN and **4b** (two doses) alone and in combination with SC. ^a^ *p* < 0.05 vs. control; ^b^ *p* < 0.05 vs. GAL; ^c^ *p* < 0.05 vs. CCN; ^d^ *p* < 0.05 vs. SC.

**Figure 3:**
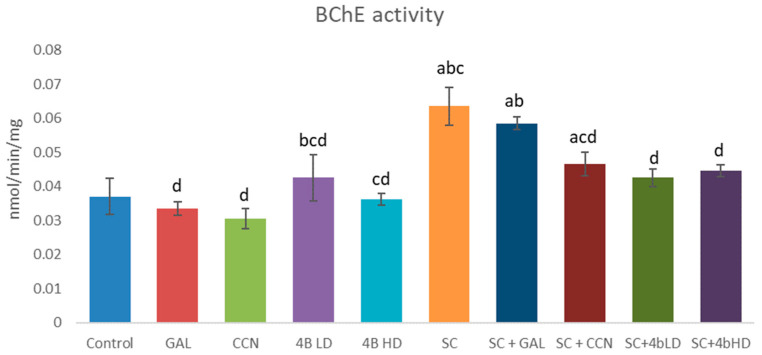
BChE activity measured for 10 min in brain homogenates from mice treated 14 days with GAL, CCN and **4b** (two doses) alone and in combination with SC. ^a^ *p* < 0.05 vs. control; ^b^ *p* < 0.05 vs. GAL; ^c^ *p* < 0.05 vs. CCN; ^d^ *p* < 0.05 vs. SC.

In the present study, the effects of GAL, CCN and **4b** alone and in combination with SC on AChE (Figure 2) and BChE (Figure 3) activity were also evaluated ex vivo. GAL, CCN and both doses of **4b** administered alone decreased AChE activity in a statistically significant manner by 11%, 21%, 20% and 25%, respectively. GAL and CCN slightly decreased the BChE activity while **4b** LD slightly increased it. Almost no change was observed on BChE activity treated by **4b** HD.

Scopolamine administered alone significantly increased both AChE and BChE activities by 32% and 70%, respectively. GAL, CCN and both doses of **4b** administered with SC reduced AChE activity by 43%, 41%, 32% and 40%, respectively, compared with the SC group (Figure 2). Regarding BChE inhibition (Figure 3), GAL did not statistically significantly change this enzyme activity, while CCN and both doses of **4b** reduced it by 27%, 33% and 30%, respectively.

## 3. Discussion

Oxidative stress is an important pathological mechanism affecting the pathogenesis of many modern socially significant diseases, including neurodegenerative ones. The brain, with its high oxygen consumption and lipid-rich content, is highly susceptible to oxidative stress. There is no doubt that the imbalance between oxidative stress and the antioxidant capacity of the human body plays a major role in the pathogenesis of AD. The search for new molecules possessing both antioxidant and cholinesterase inhibitory effects would provide great opportunities to therapeutically influence this debilitating disease.

In the present study, we evaluated the antioxidant and cholinesterase inhibitory potential of a novel GAL-CCN hybrid **4b** on SC-induced neurotoxicity, a model of Alzheimer’s dementia. CCN increased GSH levels compared with controls and GAL. These results were expected since CCN is known for its antioxidant and anti-inflammatory effects. It increases the synthesis of GSH, the main endogenous cellular protector and antioxidant [16]. CCN enters the structure of the new hybrid **4b**, which is probably the reason why the novel compound in both administered doses increased the GSH level. These results confirm the data from our previous studies where **4b**, especially at a higher dose, also increased GSH level [11].

Both doses of **4b** showed significantly higher SOD activity compared with GAL and CCN. The antioxidant enzymes in the brain are involved in the neutralization and resistance to the toxic effects of ROS. Neurons are highly sensitive to the increased mitochondrial superoxide anion (O^2•−^) [17]. The first line of defense is the SOD enzyme family, which converts (O^2•−^) to stable and easily diffusible hydrogen peroxide (H_2_O_2_) that is neutralized to water by CAT or GPx [18]. It is possible that the hybrid **4b** can specifically activate this enzyme. This fact should be verified in future experiments.

Many studies have reported that oxidative stress is one of the mechanisms with a key role in SC-induced brain damage. SC decreases the activity of SOD, CAT, glutathione S-transferase (GST) and GPx and increases the level of MDA [6]. Several antioxidant compounds, including CCN, could improve SC-induced memory impairment by attenuating oxidative stress markers [6,8], indicating a crucial role of oxidative stress in SC-induced amnesia.

The simultaneous administration of SC with GAL, CCN and **4b** improved the brain oxidative status. GAL is an AChE inhibitor and has some antioxidant properties acting as a ROS acceptor [19]. We found similar effects for GAL in our previous studies [11,20], and some other research teams have also found this [21,22].

ROS are potent tissue-damaging molecules in almost all diseases, including AD [23]. The main antioxidant mechanisms include both increased antioxidant enzyme activity and nonenzymatic mechanisms such as increased GSH levels. CAT, SOD and GPx are the main antioxidant enzymes of the brain’s defense system, by which the free radicals produced during the pathological progression of AD are removed. So, the intake of natural or synthetic antioxidants may be beneficial in slowing the progression of AD. CCN has been shown to be an antioxidant in a variety of neurodegenerative diseases, including AD [24,25]. It reduces the serum MDA concentration and has the potential to increase the total antioxidant potential (TAC). The action of CCN on markers of oxidative stress is related to its activity in scavenging radicals, chelating metals and regulating antioxidant enzymes [26]. The presence of CCN fragments in the structure of **4b** determined its significant antioxidant effect at both administered doses.

In the present study, GAL, CCN and **4b** administered alone reduced the AChE activity. Their effect on BChE was diverse: GAL and CCN slightly decreased it, **4b** LD slightly increased it and **4b** HD had no effect. SC increased both AChE and BChE activities as it was observed by others [8,27].

Regarding the BChE inhibition, the results showed that GAL did not significantly change this enzyme activity compared with the SC group, while CCN and both doses of **4b** substantially reduced the enzyme activity. In our previous study [11], using PAMPA, we found that **4b** had better blood–brain barrier (BBB) permeability compared with CCN and GAL, and thus provided a better brain bioavailability. Cholinesterase inhibition has strong clinical evidence in slowing the progression of AD. Several drugs selectively inhibit AChE, but molecules that can also inhibit BChE may provide additional benefits. As AD progresses, brain ACh concentrations may become increasingly dependent on BChE [5]. In this sense, dual enzyme inhibitors may provide a more significant efficacy than AChE-selective agents.

The increased levels of ACh in the brain are associated with the onset and development of depression [28]. Biochemically, depression is caused by a disbalance between adrenergic and cholinergic functions in favor of cholinergic dominance [29]. There is a hypothesis that the depression observed in patients with AD treated with AChE inhibitors is a side effect of the therapy and not a comorbidity [28]. Although there is still no strong evidence, there are many indications to support this hypothesis [30]. In this sense, the higher anticholinesterase effect implies a higher risk of triggering depression. We have not yet examined the prodepressant effects of **4b**, if any. The LD_50_ of **4b** in an acute toxicity test on mice is 49 mg/kg orally [11]. For comparison, the oral LD_50_s of GAL and CCN are 30 mg/kg [31] and more than 2000 mg/kg [32], respectively. The presence of the CCN fragment in the molecule of **4b** obviously decreases its toxicity. In the present study, the doses used in the in vivo experiments were 1/10 of LD_50_ for GAL, CCN and **4b** HD. The dose for **4b** LD was 1/20 of LD_50_. Even more, it was found that CCN might improve depressive and anxiety symptoms in people with depression [33]. Considering the low toxicity of **4b**, the presence of the CCN fragment in the molecule and the low administered doses, we do not expect **4b** to have a prodepressant effect, but this remains to be proven in further studies.

## 4. Materials and Methods

### 4.1. Materials

Acetylthiocholine iodide (Sigma Aldrich, Taufkirchen, Germany, Mw = 289.18, purity > 98%), beta–Nicotinamide adenine dinucleotide 2′-phosphate reduced tetrasodium salt (NADPH), Bovine serum albumin (Sigma Aldrich, Taufkirchen, Germany, Mw ≈ 66 kD, purity > 96%), Butyrylthiocholine iodide (Thermo Fisher, Kandel, Germany, Mw = 317.23, purity > 98%), Cumene hydroperoxide (Sigma Aldrich, Taufkirchen, Germany, Mw = 152.2, purity > 88%), 1-chloro-2 4-dinitrobenzene (CDNB) (Sigma Aldrich, Taufkirchen, Germany, Mw = 202.56, purity > 97%), Curcumin (BioXtract, Les Isnes, Belgium, Mw = 368.4 g/mol, purity > 98%), Dinitro-5,5 dithiodibenzoic acid (DTNB) (Sigma Aldrich, Taufkirchen, Germany, Mw = 396.35, purity > 98%), Epinephrine (Sigma Aldrich, Taufkirchen, Germany, Mw = 183.20, purity > 99%), Ethylenediaminetetraacetic acid (EDTA) (Sigma Aldrich, Taufkirchen, Germany, Mw = 292.24, purity > 99%), Galantamine HBr (Galen-N Ltd., Sofia, Bulgaria, Mw = 368.3 g/mol, purity > 98%), Glutathione oxidized (GSSG) (Sigma Aldrich, Taufkirchen, Germany, Mw = 612.63, purity > 98%), Glutathione reduced (GSH) (Sigma Aldrich, Taufkirchen, Germany, Mw = 307.32, purity > 98%), Glutathione reductase (GR) from baker’s yeast (*S. cerevisiae*) (Sigma Aldrich, Taufkirchen, Germany), Hydrogen peroxide sol. 30% (Sigma Aldrich, Taufkirchen, Germany, Mw = 34.01), Scopolamine HBr 3H_2_O (Sigma Aldrich, Taufkirchen, Germany, Mw = 438.31, purity > 98%), Thiobarbituric acid (Sigma Aldrich, Taufkirchen, Germany, Mw = 144.15 g/mol, purity > 98%), Trichloroacetic acid (Sigma Aldrich, Taufkirchen, Germany, Mw = 163.39 g/mol, purity > 99%) were used. The synthesis of **4b** was described elsewhere [8,9].

### 4.2. Animals

Forty specific pathogen-free male ICR mice (6 weeks old, 25–35 g) were used. The animals were purchased from the National Breeding Center, Sofia, Bulgaria. A minimum of 7 days of acclimatization was allowed before the start of the study. Age-appropriate standard complete commercial pelleted mouse feed and fresh drinking water were available ad libitum during the experimental period of 14 days. The animals were housed in Plexiglas cages (4 per cage) in a 12/12 light/dark cycle under standard laboratory conditions (ambient temperature 20 ± 2 °C and humidity 72 ± 4%).

Mice were housed, maintained and euthanized in accordance with relevant international rules and recommendations as outlined in the European Convention for the Protection of Vertebrate Animals Used for Experimental and Other Scientific Purposes (ETS 123).

### 4.3. Design of the Experiment

Alzheimer’s type dementia was induced by an *ip* injection of SC 3 mg/kg to male mice for a period of 14 days. To assess the effect of **4b** on SC-induced changes on brain biochemical parameters, 40 male mice were divided into ten groups with four mice in each group (n = 4) [11,20].

Group 1—control mice, treated with 0.9 % saline *po*;
Group 2—animals treated with GAL alone as a positive control (3 mg/kg *po*);
Group 3—animals treated with CCN alone as a positive control (25 mg/kg *po*);
Group 4—animals treated with **4b** alone (2.5 mg/kg *po*), (low dose, LD);
Group 5—animals treated with **4b** alone (5 mg/kg *po*) (high dose, HD);
Group 6—animals treated with SC alone (3 mg/kg *ip*);
Group 7—animals treated with GAL (3 mg/kg *po*) and SC (3 mg/kg *ip* 30 min after GAL medication);
Group 8—animals treated with CCN (25 mg/kg *po*) and SC (3 mg/kg *ip* 30 min after CCN medication);
Group 9—animals treated with **4b** (2.5 mg/kg *po*) and SC (3 mg/kg *ip* 30 min after **4b** medication);
Group 10—animals treated with **4b** (5 mg/kg *po*) and SC (3 mg/kg *ip* 30 min after **4b** medication).

All treatments were performed over 14 days. During this period, animals were observed daily for behavioral changes and signs of toxicity. On day 14, after the last treatment, the animals were euthanized in strict accordance with the rule of the Animal Ethics Committee and the adopted Directive 2010/63/EU (Directive 2010/63/EU of the European Parliament and of the Council of 22 September 2010 on the protection of animals used for scientific purposes) [34].

Brains were harvested, dried, weighed and homogenized with appropriate buffers. The protein content of brain homogenates was measured using the Lowry method [35]. The brain homogenates were used to assess cholinesterase activity (AChE and BChE) and the oxidative stress biomarkers catalase (CAT), superoxide dismutase (SOD), glutathione peroxidase (GPx), malondialdehyde (MDA) and reduced glutathione (GSH).

### 4.4. Methods

#### 4.4.1. Measurement of AChE and BChE Inhibition in Brain Homogenate

Brains were homogenized with a 0.1 M phosphate buffer, pH 7.4. Aliquots of the brain homogenates from different groups were used to measure AChE activity for 10 min via the Ellman method [36]. AChE activity was calculated and expressed as nmol/min/mg protein using a molar absorbance coefficient of 13,600 M^−1^ cm^−1^. This method is based on the formation of a yellow anion, 5,5′dithio-bis-nitrobenzoic acid, measured by absorbance at 412 nm. The reaction was initiated by adding acetylthiocholine iodide as a substrate. A measurement of BChE inhibition was performed in the same manner with butyrylthiocholine iodide used as a substrate [8]. AChE and BChE activities were calculated and expressed as nmol/min/mg protein using the molar absorption coefficient of 13,600 M^−1^ cm^−1^.

#### 4.4.2. Antioxidant Enzyme Activity Measurement

Measured amounts of brains were rinsed in ice-cold saline and minced with scissors. Furthermore, 10% homogenates were prepared in a 0.05 M phosphate buffer (pH = 7.4) and were centrifuged at 7000× *g*, and the supernatant was used for the antioxidant enzyme assay.

- Catalase Activity (CAT)

Catalase activity was assessed using the method of Aebi et al. [37]. The CAT activity was determined by monitoring the decomposition of H_2_O_2_, which was measured spectrophotometrically by the decrease in absorbance at 240 nm. Enzyme activity was calculated using a molar extinction coefficient of 0.043 mM^−1^ cm^−1^ and expressed as nM/minute/mg protein.

- Superoxide Dismutase Activity (SOD).

Superoxide dismutase activity was measured according to the method of Misra and Fridovich [38] following the spectrophotometric autoxidation of epinephrine at pH = 10.4, 30 °C, using the molar extinction coefficient of 4.02 mM^−1^ cm^−1^. The reaction was initiated by the addition of epinephrine. SOD activity was expressed as nmol of epinephrine prevented from autoxidation after the addition of the sample.

- Glutathione Peroxidase Activity (GPx).

Glutathione Peroxidase activity was measured with NADPH oxidation using a coupled reaction system consisting of glutathione, glutathione reductase and cumene hydroperoxide [39]. The reaction was initiated by adding 50 𝜇L cumene hydroperoxide (1 mg/mL) and the rate of disappearance of NADPH with time was determined by monitoring the absorbance at 340 nm. The results are expressed as nmol/min/mg protein using the molar extinction coefficient of 6.22 mM^−1^ cm^−1^.

#### 4.4.3. Measurement of Malondialdehyde (MDA) Levels in Brain Homogenate

The brains were homogenized with a 0.1 M phosphate buffer and EDTA, pH 7.4. Aliquots of the homogenates were heated for 20 min on a water bath (100 °C) with thiobarbituric acid. The amount of thiobarbituric-acid-formed reactive species (TBARS) (expressed as MDA equivalents) was measured spectrophotometrically using the method of Deby and Goutier [40] at a wavelength of 535 nm. The concentration of MDA was calculated using a molar absorption coefficient of 1.56 × 10^5^ M^−1^ cm^−1^ and is expressed in nmol/g tissue.

#### 4.4.4. Measurement of Reduced Glutathione (GSH) Levels in Brain Homogenate

Reduced Glutathione was evaluated by measuring nonprotein sulfhydryls after trichloroacetic acid (TCA) protein precipitation using the method described by Bump et al. [41]. The brains were homogenized in 5% TCA (1:10) and centrifuged for 20 min at 4000× *g*. The reaction mixture contained 0.05 mL of supernatant, 3 mL of 0.05 M phosphate buffer (pH = 8) and 0.02 mL of DTNB reagent. Absorption was determined at a wavelength of 412 nm and the results are expressed as nmol/g tissue.

#### 4.4.5. Statistical Analysis

The MEDCALC statistical program (MedCalc Software bvba, Belgium, v. 12.3) was used to analyze the data. The results are expressed as mean ± SD for four mice in each group. The significance of the data was assessed with a nonparametric Mann–Whitney *U* test. Values of *p* < 0.05 were considered statistically significant.

## 5. Conclusions

The results in the present study expand our knowledge about the multitarget action of the hybrid **4b**. It was found that this compound completely protects and eliminates the toxic effects of SC administered in vivo. Moreover, this effect was observed for both AChE and BChE. The protection of **4b** against BChE is a novelty, the mechanism of which remains to be elucidated, because apparently it is not due to a direct interaction with the enzyme. The antioxidant activity of the novel compound was recorded here as a reactivation of the antioxidant enzymes CAT, SOD and GPx, injured by SC administration. In this study again, **4b** outperformed its parents GAL and CCN. Combining fragments from GAL and CCN provides not just a sum of their activities but adds a new surplus value to the novel hybrid, making it promising for further experimental studies in the field of neurodegenerative processes.

## Figures and Tables

**Table 1:** Oxidative stress biomarkers for GAL, CCN and **4b** (two doses) alone and in combination with SC. Results are expressed as mean ± SD (n = 4). The significance of the data was assessed using the nonparametric Mann–Whitney *U* test. Values of *p* ≤ 0.05 were considered statistically significant.

Parameters	Control	GAL	CCN	4b LD	4b HD	SC	SC + GAL	SC + CCN	SC + 4b LD	SC + 4b HD
CAT nmol/mg/min	8.50 ± 0.41	7.70 ± 0.42 ^d^	9.35 ± 0.68 ^d^	7.80 ± 0.32 ^cd^	7.10 ± 0.70 ^cd^	5.35 ± 0.52 ^abc^	7.20 ± 0.43 ^d^	8.55 ± 0.34 ^d^	8.15 ± 0.28 ^d^	8.40 ± 0.36 ^d^
SOD nmol/mg/min	0.35 ± 0.05	0.31 ± 0.04 ^d^	0.34 ± 0.01 ^d^	0.44 ± 0.03 ^bcd^	0.41 ± 0.03 ^bcd^	0.22 ± 0.03 ^abc^	0.31 ± 0.02 ^d^	0.34 ± 0.01 ^d^	0.33 ± 0.01 ^d^	0.34 ± 0.02 ^d^
GPx nmol/mg/min	0.43 ± 0.01	0.39 ± 0.03 ^d^	0.40 ± 0.04 ^d^	0.44 ± 0.03 ^d^	0.46 ± 0.02 ^d^	0.21 ± 0.02 ^abc^	0.34 ± 0.03 ^ad^	0.39 ± 0.05 ^d^	0.41 ± 0.03 ^d^	0.44 ± 0.02 ^d^
GSH nmol/g tissue	1.59 ± 0.03	1.73 ± 0.07 ^d^	2.04 ± 0.12 ^abd^	2.06 ± 0.07 ^abd^	2.22 ± 0.08 ^abd^	1.09 ± 0.13 ^abc^	1.66 ± 0.12	1.83 ± 0.03 ^cd^	1.44 ± 0.08 ^d^	1.59 ± 0.04 ^d^
MDA nmol/g tissue	1.41 ± 0.27	1.75 ± 0.13 ^d^	1.45 ± 0.09 ^d^	1.62 ± 0.30 ^d^	1.76 ± 0.12 ^d^	2.39 ± 0.1 ^abc^	1.79 ± 0.08 ^d^	1.64 ± 0.05 ^d^	1.76 ± 0.03 ^d^	1.65 ± 0.16 ^d^

Abbreviations: GAL, galantamine; CCN, curcumin; **4b** LD, **4b** low dose (2.5 mg/kg); **4b** HD, **4b** high dose (5 mg/kg); SC, scopolamine; ^a^ *p* < 0.05 vs. control; ^b.^*p* < 0.05 vs. GAL; ^c^ *p* < 0.05 vs. CCN; ^d^ *p* < 0.05 vs. SC.

## Data Availability

Not applicable.
